# Drug Overdose Mortality Among People Experiencing Homelessness, 2003 to 2018

**DOI:** 10.1001/jamanetworkopen.2021.42676

**Published:** 2022-01-07

**Authors:** Danielle R. Fine, Kirsten A. Dickins, Logan D. Adams, Denise De Las Nueces, Karen Weinstock, Joseph Wright, Jessie M. Gaeta, Travis P. Baggett

**Affiliations:** 1 Division of General Internal Medicine, Department of Medicine, Massachusetts General Hospital, Boston; 2 Harvard Medical School, Boston, Massachusetts; 3 Munn Center for Nursing Research, Massachusetts General Hospital, Boston; 4 Institute for Research, Quality, and Policy in Homeless Health Care, Boston Health Care for the Homeless Program, Boston, Massachusetts; 5 Section of General Internal Medicine, Boston University School of Medicine, Boston, Massachusetts; 6 University of Miami School of Medicine, Miami, Florida

## Abstract

**Importance:**

Despite high rates of drug overdose death among people experiencing homelessness, patterns in drug overdose mortality, including the types of drugs implicated in overdose deaths, remain understudied in this population.

**Objective:**

To describe the patterns in drug overdose mortality among a large cohort of people experiencing homelessness in Boston vs the general adult population of Massachusetts and to evaluate the types of drugs implicated in overdose deaths over a continuous 16-year period of observation.

**Design, Setting, and Participants:**

This cohort study analyzed adults aged 18 years or older who received care at Boston Health Care for the Homeless Program (BHCHP) between January 1, 2003, and December 31, 2017. Individuals were followed up from the date of their initial BHCHP encounter during the study period until the date of death or December 31, 2018. Data were analyzed from December 1, 2020, to June 6, 2021.

**Main Outcomes and Measures:**

Drug overdose deaths and the types of drugs involved in each overdose death were ascertained by linking the BHCHP cohort to the Massachusetts Department of Public Health death records.

**Results:**

In this cohort of 60 092 adults experiencing homelessness (mean [SD] age at entry, 40.4 [13.1] years; 38 084 men [63.4%]), 7130 individuals died by the end of the study period. A total of 1727 individuals (24.2%) died of a drug overdose. Of the drug overdose decedents, 456 were female (26.4%), 194 were Black (11.2%), 202 were Latinx (11.7%), and 1185 were White (68.6%) individuals, and the mean (SD) age at death was 43.7 (10.8) years. The age- and sex-standardized drug overdose mortality rate in the BHCHP cohort was 278.9 (95% CI, 266.1-292.3) deaths per 100 000 person-years, which was 12 times higher than the Massachusetts adult population. Opioids were involved in 91.0% of all drug overdose deaths. Between 2013 and 2018, the synthetic opioid mortality rate increased from 21.6 to 327.0 deaths per 100 000 person-years. Between 2004 and 2018, the opioid-only overdose mortality rate decreased from 117.2 to 102.4 deaths per 100 000 person-years, whereas the opioid-involved polysubstance mortality rate increased from 44.0 to 237.8 deaths per 100 000 person-years. Among opioid-involved polysubstance overdose deaths, cocaine-plus-opioid was the most common substance combination implicated throughout the study period, with Black individuals having the highest proportion of cocaine-plus-opioid involvement in death (0.72 vs 0.62 in Latinx and 0.53 in White individuals; *P* < .001).

**Conclusions and Relevance:**

In this cohort study of people experiencing homelessness, drug overdose accounted for 1 in 4 deaths, with synthetic opioid and polysubstance involvement becoming predominant contributors to mortality in recent years. These findings emphasize the importance of increasing access to evidence-based opioid overdose prevention strategies and opioid use disorder treatment among people experiencing homelessness, while highlighting the need to address both intentional and unintentional polysubstance use in this population.

## Key Points

**Question:**

What are the temporal patterns in drug overdose mortality among people experiencing homelessness?

**Findings:**

In this cohort study of 60 092 individuals experiencing homeless in Boston, Massachusetts, drug overdose accounted for 1 in 4 deaths. Mortality patterns over time showed a substantial increase in synthetic opioid overdose mortality and a predominance of opioid-involved polysubstance overdose deaths in recent years.

**Meaning:**

The findings suggest that ongoing efforts to improve access to evidence-based opioid overdose prevention strategies and opioid use disorder treatment are needed for people experiencing homelessness.

## Teaser

This cohort study describes the death rates and drug types involved in fatal drug overdoses in the past 15 years among individuals experiencing homelessness in Boston, Massachusetts.

## Introduction

Drug overdose mortality has increased substantially over the past 20 years in the United States.^1^ People experiencing homelessness have been disproportionately affected by this public health crisis, with drug overdose emerging as a leading cause of death at rates up to 30-fold higher than in the US general population.^2,3,4^ Despite these circumstances, temporal patterns in drug overdose mortality and the types of drugs implicated in overdose deaths remain understudied in this population.

In the US general population, drug overdose patterns have demonstrated substantial shifts in the types of drugs implicated in deaths over the past 2 decades. Prescription opioids were initially the predominant drugs involved in overdose-related deaths before a shift to heroin in 2010.^5^ However, between 2013 and 2019, overdose deaths involving synthetic opioids, such as illicitly manufactured fentanyl, increased by more than 11-fold.^5^ Polysubstance overdose mortality rates, particularly involving synthetic opioids combined with cocaine and psychostimulants, have also been increasing in recent years.^5,6^

Public health patterns in the general population are often magnified in people experiencing homelessness, most of whom have high levels of medical and psychosocial comorbidities and are overrepresented in Black and Latinx individuals.^7^ Understanding drug overdose patterns in this population over time is crucial to advancing health equity across racially, medically, and socioeconomically diverse communities and the health care systems that serve them. The primary objectives of this study were to (1) describe the patterns in drug overdose mortality among a large cohort of people experiencing homelessness in Boston compared with the general adult population of Massachusetts and (2) evaluate the types of drugs implicated in overdose deaths over a continuous 16-year period of observation. Because previous studies found that mortality patterns by race and ethnicity in the homeless community do not always reflect those seen in the larger population,^3,8^ we also examined drug overdose mortality patterns by race and ethnicity. We believe a comprehensive assessment of drug overdose mortality and the evolving types of drugs involved in overdose deaths will help to inform policy decisions and clinical practice for people experiencing homelessness.

## Methods

### Participants and Setting

We created a retrospective cohort of adults (aged ≥18 years) who had 1 or more encounters at Boston Health Care for the Homeless Program (BHCHP) between January 1, 2003, and December 31, 2017. The Mass General Brigham Institutional Review Board approved this cohort study and waived the informed consent requirement because the study posed minimal risk to the individuals involved. We followed the Strengthening the Reporting of Observational Studies in Epidemiology (STROBE) reporting guideline.^9^

Boston Health Care for the Homeless Program provides comprehensive health care services to more than 11 000 patients annually at more than 40 locations in the greater Boston, Massachusetts area.^10^ To enroll in care at BHCHP, individuals must be currently unhoused; however, some individuals may elect to continue receiving care at BHCHP after they have obtained housing. Internal BHCHP data suggest that approximately 16% to 20% of patients are housed during any given year, although many of these dwellings are in unstable settings that change over time. Thus, this study cohort included individuals who were living on the street, in shelters, or in unstable housing. Individuals were followed up from the date of their initial BHCHP encounter during the study period until the date of death or December 31, 2018.

Race and ethnicity data were obtained from BHCHP or CDC WONDER (Centers for Disease Control and Prevention Wide-ranging Online Data for Epidemiologic Research) records and were either self-identified or identified based on observation. The following race and ethnicity categories were analyzed: American Indian or Alaska Native, Asian or Pacific Islander, Black, Latinx, White, or other (including >1 race and unknown).

### Vital Status

We identified deaths by cross-linking the BHCHP cohort to the Massachusetts Department of Public Health death records from January 1, 2003, to December 31, 2018, using Match*Pro, version 1.6.3 (IMS Inc). Match*Pro is a probabilistic record-linkage software program based on the Fellegi-Sunter method that computes linkage probability weights for possible record pairs.^11^ The linkage procedure used first and last name, date of birth, and Social Security number. Two of us (D.R.F. and K.A.D.) independently manually reviewed each possible record pair that achieved a probability score of 0.45 or higher. A pair was considered a true linkage if it matched on 1 or more of the following National Death Index criteria: (1) Social Security number; (2) first and last name, and month and year of birth (plus or minus 1 year); or (3) first and last name, and month and day of birth.^12^ The 2 investigators achieved high concordance and interrater reliability (κ > 0.99). A third investigator adjudicated the discrepancies found. To draw mortality comparisons with the adult population of Massachusetts, we obtained mortality data for the state from the CDC WONDER Underlying Cause of Death files for January 1, 2003, to December 31, 2018.^1^

### Cause of Death

We used the *ICD-10* (*International Statistical Classification of Diseases and Related Health Problems, Tenth Revision*) codes for the underlying cause of death to define drug overdose deaths^13^ (eTable 1 in the Supplement). We used the multiple cause of death field *ICD-10* T codes to identify the types of drugs involved in each drug overdose death^14,15,16,17^ (eTable 1 in the Supplement). A drug overdose death could involve more than 1 type of drug. T codes are based on toxicological specimens that were obtained during autopsy, from stand-alone postmortem testing, or from antemortem collection during a hospitalization.^18^ In this cohort, autopsy was performed in 1115 (64.6%) of the 1727 drug overdose decedents, with only 21 overdose decedents (1.2%) missing drug-specific information; these 21 individuals were excluded from drug-specific mortality analyses.

### Statistical Analysis

We plotted age- and sex-standardized drug overdose mortality rates over time for the BHCHP cohort and the Massachusetts cohort. We applied indirect standardization, a procedure that calculates the expected mortality rate for an index population, using specific mortality rates stratified by the adjustment variables from a reference population. We used the aggregated Massachusetts adult population from January 1, 2004, to December 31, 2018, as the reference population to calculate age- and sex-standardized yearly mortality rates. We were unable to standardize annual rates by race and ethnicity because of the confidentiality constraints within CDC WONDER (data are suppressed for rows of <10 people). We excluded 2003 data from temporal pattern analyses because insufficient person-years of observation in the BHCHP cohort created highly uncertain mortality estimates for that particular year. However, 2003 data were included in all analyses that were aggregated across years. We presented mortality rates as the number of deaths per 100 000 person-years and fit 95% CIs using exact methods for standardized mortality ratios.

We examined temporal patterns in the drugs implicated in overdose deaths for the BHCHP cohort. Specifically, we evaluated crude drug overdose mortality rates over time by (1) major drug categories involved in death; (2) type of opioids involved in opioid overdose deaths; and (3) polysubstance vs single substance involvement in death, with a focus on deaths that involved opioids. We defined polysubstance-involved overdose mortality as deaths that implicated 2 or more categories of substances, including alcohol. Conversely, single substance–involved overdose mortality was defined as deaths that implicated only 1 drug category and not alcohol.

To assess the variations in drug overdose mortality patterns by race and ethnicity, we performed 3 additional analyses. First, we compared the race and ethnicity–stratified, age- and sex-standardized mortality rates between the BHCHP and Massachusetts cohorts using aggregated data for all study years. We calculated the standardized mortality rate ratios by dividing the standardized mortality rates in the BHCHP cohort by the standardized mortality rates in the Massachusetts cohort. Second, we plotted crude drug overdose mortality rates by race and ethnicity in the BHCHP cohort from 2004 through 2018. Third, we assessed opioid-involved polysubstance overdose mortality rates by race and ethnicity over the entire study period. We performed Fisher exact tests to evaluate the differences by race and ethnicity in the proportions of deaths in major polysubstance categories. A 2-sided *P* < .05 was considered to be statistically significant.

To present a comprehensive analysis of drug overdose mortality patterns among people experiencing homelessness, we performed these same 3 analyses by sex as well. We used RStudio, version 1.2.5033 (RStudio) and Microsoft Excel (Microsoft Corp) for all analyses, which were conducted from December 1, 2020, to June 6, 2021.

## Results

### Cohort Characteristics

A total of 60 092 adults experiencing homelessness who were enrolled to receive care at BHCHP were followed up for a median duration of 8.7 years, yielding 520 429.6 person-years of observation. This BHCHP cohort had a mean (SD) age at entry of 40.4 (13.1) years and was composed of 38 084 men (63.4%) and 22 008 women (36.6%). Of these patients, 15 928 were of Black (26.5%), 10 773 of Latinx (17.9%), and 26 364 of White (43.9%) race and ethnicity (Table).

**Table.** Characteristics of Adults Enrolled in the Boston Health Care for the Homeless Program (BHCHP) Cohort From 2003 to 2017

Characteristic	No. (%)		
	BHCHP cohort	Decedents (n = 7130)	
		Drug overdose	Other causes of death
No. of enrolled adults	60 092	1727	5403
Age at enrollment, mean (SD), y	40.4 (13.1)	38.5 (10.3)	50.9 (11.6)
Sex			
Male	38 084 (63.4)	1271 (73.6)	4258 (78.8)
Female	22 008 (36.6)	456 (26.4)	1145 (21.2)
Race and ethnicity^a^			
American Indian or Alaska Native	316 (0.5)	6 (0.3)	27 (0.5)
Asian or Pacific Islander	749 (1.2)	6 (0.3)	35 (0.6)
Black	15 928 (26.5)	194 (11.2)	1306 (24.2)
Latinx	10 773 (17.9)	202 (11.7)	508 (9.4)
White	26 364 (43.9)	1185 (68.6)	3201 (59.2)
Other^b^	5962 (9.9)	134 (7.8)	326 (6.0)
Age at death, mean (SD), y	NA	43.7 (10.8)	56.9 (12.2)
Autopsy performed	NA	1115 (64.6)	1301 (24.1)
Place of death			
Hospital	NA	448 (25.9)	2568 (47.5)
Dead on arrival to hospital	NA	231 (13.4)	126 (2.3)
Residence	NA	376 (21.8)	532 (9.8)
Nursing home or assisted living	NA	433 (25.1)	1464 (27.1)
Hospice	NA	3 (0.2)	544 (10.1)
Other or unknown	NA	236 (13.7)	169 (3.1)

Abbreviation: NA, not applicable.

^a^ Race and ethnicity data were obtained from BHCHP records and were either self-identified or identified based on observation.

^b^ Other race and ethnicity included more than 1 race and unknown.

A total of 7130 patients died by the end of the study period. Drug overdose accounted for 1727 deaths (24.2%). Among those who died from a drug overdose, 1271 (73.6%) were male, 456 were female (26.4%), 194 were Black (11.2%), 202 were Latinx (11.7%), and 1185 were White (68.6%) individuals. The mean (SD) age at death was 43.7 (10.8) years among those who died of drug overdose compared with 56.9 (12.2) years among those who died of other causes.

### Drug Overdose Mortality in BHCHP vs Massachusetts Cohort 

**Figure 1.**
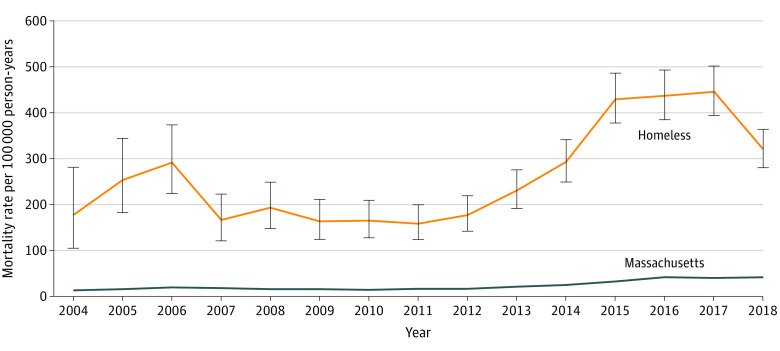
Drug Overdose Mortality in the Boston Health Care for the Homeless Program (BHCHP) Cohort vs the Massachusetts Adult Population From 2004 to 2018 Age and sex indirect standardization was applied each year to both the BHCHP cohort (orange line) and the Massachusetts population (blue line) using the aggregated 2004 to 2018 Massachusetts adult population as the reference population. Error bars represent 95% CIs.

The age- and sex-standardized drug overdose mortality rate in the BHCHP cohort was 12 times higher than in the Massachusetts general population (278.9 [95% CI, 266.1-292.3] vs 23.2 [95% CI, 22.8-23.5] deaths per 100 000 person-years). Between 2004 and 2018, the drug overdose mortality rate increased from 177.6 to 321.1 deaths per 100 000 person-years in the BHCHP cohort (Figure 1).

### Drugs Implicated in Overdose Deaths

**Figure 2.**
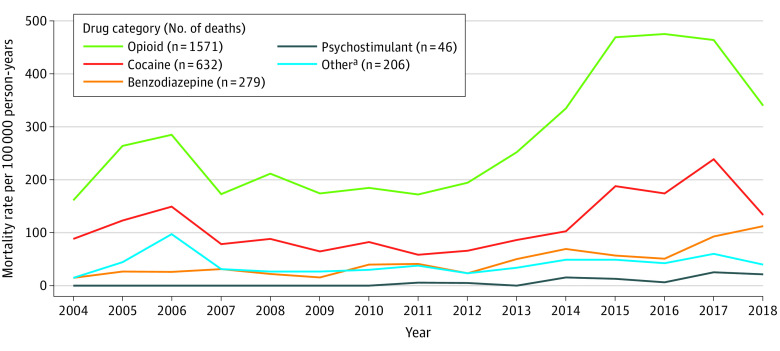
Drug Overdose Mortality in the Boston Health Care for the Homeless Program Cohort by Type of Drug Involved From 2004 to 2018 ^a^Other includes antidepressants, antipsychotics, barbiturates, and other neuroleptics and sedatives. Drug categories are not mutually exclusive.

**Figure 3.**
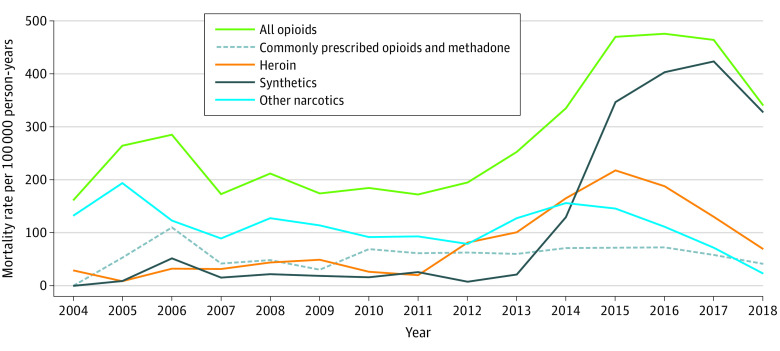
Opioid-Involved Mortality in the Boston Health Care for the Homeless Program Cohort by Type of Opioid Involved From 2004 to 2018 Opioid categories are not mutually exclusive.

Opioids were the leading drug category involved in overdose deaths (1571 of 1727 [91.0%]), followed by cocaine (632 [36.6%]) and benzodiazepines (279 [16.2%]). Between 2004 and 2018, the opioid-involved overdose mortality rate increased from 161.2 to 340.2 deaths per 100 000 person-years (Figure 2). The composition of opioid-involved deaths changed over time. Between 2013 and 2018, the synthetic opioid mortality rate increased from 21.6 to 327.0 deaths per 100 000 person-years (Figure 3). In 2018, synthetic opioids were involved in 96.1% of all opioid-involved deaths (199 of 207) compared with 0% (0 of 11) in 2004.

**Figure 4.**
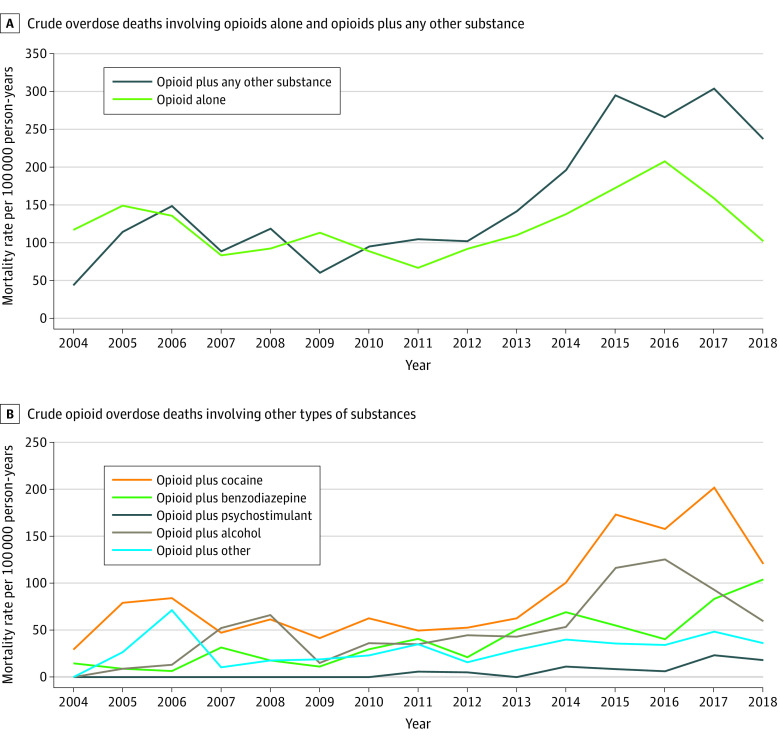
Opioid-Involved Polysubstance Overdose Mortality in the Boston Health Care for the Homeless Program Cohort From 2004 to 2018 Categories of substance combination involvement are not mutually exclusive.

Opioids were involved in 93.6% of all polysubstance-involved deaths (923 of 986) (eTable 2 in the Supplement). Between 2004 and 2018, the opioid-only mortality rate decreased from 117.2 to 102.4 deaths per 100 000 person-years, whereas the opioid-involved polysubstance mortality rate increased from 44.0 to 237.8 deaths per 100 000 person-years (Figure 4). In 2018, opioid-involved polysubstance deaths accounted for 64.6% of all drug overdose deaths (144 of 223). Cocaine-plus-opioid was the most common substance combination implicated in opioid-involved polysubstance deaths across the study period (Figure 4).

### Drug Overdose Mortality Patterns by Race and Ethnicity

When comparing the aggregated drug overdose mortality rate in the BHCHP cohort vs the Massachusetts cohort, the standardized mortality rate ratio was the lowest in Black individuals (5.5; 95% CI, 4.8-6.3) and the highest in Asian or Pacific Islander individuals (44.3; 95% CI, 18.0-92.2) (eFigure 1 in the Supplement). Between 2004 and 2018, the drug overdose mortality rate increased from 128.4 to 148.0 deaths per 100 000 person-years among Black individuals, from 77.2 to 245.6 deaths per 100 000 person-years among Latinx individuals, and from 367.9 to 557.1 deaths per 100 000 person-years among White individuals (eFigure 2 in the Supplement).

Substance combinations in opioid-involved polysubstance mortality differed by race and ethnicity. Black individuals had the highest proportion of cocaine-plus-opioid (0.72 vs 0.62 in Latinx and 0.53 in White individuals; *P* < .001) and alcohol-plus-opioid (0.44 vs 0.41 in Latinx and 0.33 in White individuals; *P* = .009) involvement in overdose death, whereas White individuals had the highest proportion of benzodiazepine-plus-opioid involvement (0.32 vs 0.07 in Black and 0.20 in Latinx individuals; *P* < .001) (eTable 3 in the Supplement).

### Drug Overdose Mortality Patterns by Sex

When comparing the aggregated drug overdose mortality rate in the BHCHP cohort vs the Massachusetts cohort, the standardized mortality rate ratio among men was 11.3 (95% CI, 10.7-11.9) and 16.2 (95% CI, 14.8-17.8) among women (eFigure 3 in the Supplement). Across the study period, drug overdose mortality rates were higher among men (eFigure 4 in the Supplement). Drug combinations in opioid-involved polysubstance mortality differed by sex. Men had a higher proportion of alcohol-plus-opioid involvement in overdose death compared with women (0.37 vs 0.25; *P* = .002), whereas women had a higher proportion of benzodiazepine-plus-opioid involvement compared with men (0.34 vs 0.25; *P* = .009) (eTable 4 in the Supplement).

## Discussion

This comprehensive, large-scale cohort study of drug overdose mortality patterns among adults experiencing homelessness presents 4 major findings. First, drug overdose mortality increased 81% between 2004 and 2018, with a standardized mortality rate that was 12 times higher than in the Massachusetts cohort when aggregated across all years. Second, synthetic opioid–involved mortality increased sharply during the study period, with synthetic opioids almost universally present in opioid-involved overdose deaths by 2018. Third, polysubstance opioid overdose deaths have surpassed opioid–only overdose deaths. Fourth, drug overdose mortality patterns varied by race and ethnicity.

As has been reported in previous studies that evaluated patterns in drug overdose mortality among people experiencing homelessness,^19,20^ drug overdose was the leading cause of death in this population, with drug overdoses accounting for 1 in every 4 deaths. In contrast to these previous studies, which consisted of cohorts in California and had a predominance of psychostimulant-involved deaths, the present study found that opioids were involved in 91.0% of all overdose deaths. Although the overdose mortality rate in the BHCHP cohort declined in 2018 (the last year for which mortality data were available at the time of the analysis), data from the US general population suggested that this decrease could be short lived. In 2018, the US experienced a reduction in drug overdose mortality for the first time in more than 3 decades^21^; however, these rates started to increase again in 2019^22^ and accelerated throughout 2020 during the COVID-19 pandemic.^23^

In the BHCHP cohort, synthetic opioid mortality surged by more than 1400% between 2013 and 2018. Opioid overdose mortality became nearly synonymous with synthetic opioid overdose mortality in this population. Synthetic opioids, such as illegally manufactured fentanyl, were involved in 96.1% of opioid-related deaths in 2018. Because opioids were implicated in nearly all drug overdose deaths, synthetic opioids became predominant in drug overdose mortality in the BHCHP cohort.

Polysubstance overdose deaths involving opioids increased in prevalence during the study period, accounting for 2 of every 3 drug overdose deaths (64.6%) in 2018. This pattern has been reported in the US general population, although with mortality rates that were substantially lower than those in the BHCHP cohort.^5,16,22^ As previously found in the Massachusetts population, most opioid-involved polysubstance deaths in the BHCHP cohort included cocaine.^24^

Drug overdose mortality was higher among all racial and ethnic groups in the BHCHP cohort than in the Massachusetts cohort. However, the relative difference in mortality was substantially larger among White individuals than that observed among Black and Latinx individuals. Similar findings have been observed in previous mortality studies of people experiencing homelessness^3,8^ and may reflect the underlying mechanisms of structural racism. The path toward homelessness often differs according to a person’s race and ethnicity, with factors such as discriminatory housing policies, unequal economic opportunities, and disproportionate involvement in the criminal-legal system playing an outsized role for Black and Latinx individuals.^25^ Conversely, mental illness and substance use disorders are more prevalent factors associated with homelessness among White individuals,^26^ potentially contributing to more adverse substance use disorder–related outcomes. In addition, limited access to opioid overdose prevention programs and lack of long-term retention in addiction treatment among housed Black and Latinx individuals^27,28,29,30,31,32,33,34^ may further contribute to the reduced disparity between these individuals and their unhoused counterparts as compared with housed and unhoused White individuals. Between 2010 and 2017, the mortality patterns by race and ethnicity in the BHCHP cohort reflected those observed in the US general population,^35^ with Black and Latinx individuals experiencing faster relative increases in drug overdose mortality compared with White persons.

We believe the findings of this study have important policy and clinical implications. The substantial increase in opioid overdose deaths, particularly synthetic opioid–involved deaths, highlights the need to improve access to evidence-based opioid use disorder treatment and overdose prevention strategies, such as supervised injection facilities,^36^ fentanyl testing strips,^37^ and naloxone distribution,^38^ among people experiencing homelessness. During the years under study, BHCHP provided substance use disorder treatment primarily in office-based settings.^39^ To better reach this population, addiction and harm-reduction services may need to be provided in nontraditional settings, such as mobile addiction vans, shelters, supportive housing, and other places where homeless-experienced individuals with substance use disorders congregate.^39,40^ Furthermore, given the high burden of comorbid mental health disease in this population,^41^ expanding access to behavioral health services may improve outcomes.

The predominance of opioid-involved polysubstance overdose deaths reflects a nationwide shift in drug overdose patterns.^5,16,22^ Clinicians who serve people experiencing homelessness should take a broad approach to assessing for all types of drug use to appropriately tailor treatment. Although evidence-based treatment options for other drug use disorders are somewhat limited, contingency management has been found to be an effective treatment strategy for both opioid use disorder and stimulant use disorder.^42,43^ Polysubstance use can be intentional, but individuals who use drugs are sometimes inadvertently or unknowingly exposed to other drugs, particularly synthetic opioids. The same harm-reduction strategies mentioned earlier, in addition to a low-barrier, safer drug supply^44^ and educational efforts that address unintentional opioid exposure, need to be accessible to all homeless-experienced individuals who use drugs.

### Limitations

This study has several limitations. The findings may not be generalizable to people experiencing homelessness in Boston who sought care outside of BHCHP or who do not engage with the health care system at all. Although it is difficult to accurately characterize the general homeless population, limited demographic data from the City of Boston’s annual homeless census suggest that the BHCHP cohort’s racial and ethnic breakdown is similar to that of Boston’s general homeless population.^45^ In addition, drug overdose patterns differ by geographic region; thus, the findings of this study may not be generalizable to nonurban or non–East Coast settings.

Death categorization in death certificates can lack sensitivity and specificity. In the US general population, only 8% of deceased individuals undergo an autopsy,^46^ with up to 25% of all drug overdose deaths missing drug-specific information in the multiple cause of death fields.^47^ In the BHCHP cohort, however, the autopsy rate among drug overdose decedents was 64.6%, with only 1.2% missing drug-specific information, thereby providing some reassurance that the cause of death information was more complete than the information available for the US general population. In addition, it is likely that the drugs involved in overdose deaths were sometimes coded into the broadest category available, limiting the accuracy of drug-specific estimates. Furthermore, the lack of specific drug codes for fentanyl and methamphetamine made it difficult to accurately quantify these drug-specific mortality rates. Many of these limitations are inherent in any study of drug overdose mortality and are not unique to the BHCHP cohort.

## Conclusions

In this large, Boston-based cohort of people experiencing homelessness, drug overdose mortality was 12 times higher than in the general Massachusetts population and accounted for approximately 1 in every 4 deaths. Opioids were implicated in most drug overdose deaths, with synthetic opioids and polysubstance involvement becoming predominant in recent years. These findings highlight the need to improve access to evidence-based opioid overdose prevention strategies and opioid use disorder treatment and to address both intentional and unintentional polysubstance use in this population. Variations in overdose patterns by race and ethnicity emphasize the need to remove structural barriers to addiction care.

## Notes

Supplement. **eTable 1.** ICD-10 Codes Defining Drug Overdose and the Types of Drugs Implicated in Deaths **eTable 2.** Polysubstance Drug Overdose Mortality by Drug Categories Involved in Death in the Boston Health Care for the Homeless Cohort From 2004 to 2018 **eFigure 1.** Drug Overdose Mortality in the Boston Health Care for the Homeless Cohort, Stratified by Race and Ethnicity **eFigure 2.** Crude Drug Overdose Mortality in the Boston Health Care for the Homeless Cohort From 2004 to 2018, Stratified by Race and Ethnicity **eTable 3.** Opioid-Involved Overdose Mortality by Race and Ethnicity in the Boston Health Care for the Homeless Cohort **eFigure 3.** Drug Overdose Mortality in a Cohort of Homeless-Experienced Adults, Stratified by Sex **eFigure 4.** Crude Drug Overdose Mortality in the Boston Health Care for the Homeless Cohort From 2004 to 2018, Stratified by Sex **eTable 4.** Opioid-Involved Overdose Mortality by Sex in the Boston Health Care for the Homeless Cohort
