# Supplementary material for: Drug Overdose Mortality Among People Experiencing Homelessness, 2003 to 2018
Source: JAMA Netw Open. 2022 Jan 7;5(1):e2142676. doi: 10.1001/jamanetworkopen.2021.42676 (PMC8742197; doi:10.1001/jamanetworkopen.2021.42676)
Supplement: Supplement. — eTable 1. ICD-10 Codes Defining Drug Overdose and the Types of Drugs Implicated in Deaths eTable 2. Polysubstance Drug Overdose Mortality by Drug Categories Involved in Death in the Boston Health Care for the Homeless Cohort From 2004 to 2018 eFigure 1. Drug Overdose Mortality in the Boston Health Care for the Homeless Cohort, Stratified by Race and Ethnicity eFigure 2. Crude Drug Overdose Mortality in the Boston Health Care for the Homeless Cohort From 2004 to 2018, Stratified by Race and Ethnicity eTable 3. Opioid-Involved Overdose Mortality by Race and Ethnicity in the Boston Health Care for the Homeless Cohort eFigure 3. Drug Overdose Mortality in a Cohort of Homeless-Experienced Adults, Stratified by Sex eFigure 4. Crude Drug Overdose Mortality in the Boston Health Care for the Homeless Cohort From 2004 to 2018, Stratified by Sex eTable 4. Opioid-Involved Overdose Mortality by Sex in the Boston Health Care for the Homeless Cohort [file jamanetwopen-e2142676-s001.pdf]

Supplementary Online Content

Fine DR, Dickins KA, Adams LD, et al. Drug overdose mortality among people experiencing homelessness, 2003 to 2018. *JAMA Netw Open*. 2022;5(1):e2142676. doi:10.1001/jamanetworkopen.2021.42676

eTable 1. ICD-10 Codes Defining Drug Overdose and the Types of Drugs Implicated in Deaths

eTable 2. Polysubstance Drug Overdose Mortality by Drug Categories Involved in Death in the Boston Health Care for the Homeless Cohort From 2004 to 2018

eFigure 1. Drug Overdose Mortality in the Boston Health Care for the Homeless Cohort, Stratified by Race and Ethnicity

eFigure 2. Crude Drug Overdose Mortality in the Boston Health Care for the Homeless Cohort From 2004 to 2018, Stratified by Race and Ethnicity

eTable 3. Opioid-Involved Overdose Mortality by Race and Ethnicity in the Boston Health Care for the Homeless Cohort

eFigure 3. Drug Overdose Mortality in a Cohort of Homeless-Experienced Adults, Stratified by Sex

eFigure 4. Crude Drug Overdose Mortality in the Boston Health Care for the Homeless Cohort From 2004 to 2018, Stratified by Sex

eTable 4. Opioid-Involved Overdose Mortality by Sex in the Boston Health Care for the Homeless Cohort

This supplementary material has been provided by the authors to give readers additional information about their work.

© 2022 Fine DR et al. *JAMA Network Open.*

eTable 1. ICD-10 Codes Defining Drug Overdose and the Types of Drugs Implicated in Deaths

Category	ICD-10 Code
Drug Overdose	
Accidental	X40-X44
Intentional	X60-X64
Assault	X85
Undetermined intent	Y10-Y14
Opioids	
Heroin	T40.1
Commonly prescribed opioids and methadone	T40.2, T40.3
Synthetics	T40.4
Other and unspecific narcotics	T40.6
Cocaine	T40.5
Psychostimulants with misuse potential	T43.6
Benzodiazepines	T42.4
Other	
Antidepressants	T43.0-43.2
Antipsychotics	T43.3-43.5
Barbiturates	T42.3
Other neuroleptics and sedatives	T42.5-T42.7

© 2022 Fine DR et al. *JAMA Network Open.*

eTable 2. Polysubstance Drug Overdose Mortality by Drug Categories Involved in Death in the Boston Health Care for the Homeless Cohort From 2004 to 2018

	Polysubstance-involved		Opioid-involved[a]		Cocaine-involved[a]		Benzodiazepine-involved[a]		Psychostimulant-involved[a]	
**Year**	**n**	**Crude rate[b] (95% CI)**	**n (%)**	**Crude rate[b] (95% CI)**	**n (%)**	**Crude rate[b] (95% CI)**	**n (%)**	**Crude rate[b] (95% CI)**	**n (%)**	**Crude rate[b] (95% CI)**
2004	3	44.0 (9.1, 128.5)	3 (100)	44.0 (9.1, 128.5)	2 (67)	29.3 (3.5, 105.9)	1 (33)	14.7 (0.4, 81.6)	0 (0)	0 (0, 54.0)
2005	15	132.1 (73.9, 217.9)	13 (87)	114.45 (61.0, 195.8)	10 (67)	88.1 (42.2, 161.9)	2 (13)	17.6 (2.1, 63.6)	0 (0)	0 (0, 32.5)
2006	28	181.4 (120.6, 262.4)	23 (82)	149.0 (94.5, 223.6)	16 (57)	103.7 (59.3, 168.4)	3 (11)	19.4 (4.0, 56.8)	0 (0)	0 (0, 23.9)
2007	20	104.8 (64.0, 161.9)	17 (85)	89.1 (51.9, 142.7)	11 (55)	57.7 (28.8, 103.2)	6 (30)	31.4 (11.5, 68.4)	0 (0)	0 (0, 19.3)
2008	32	141.0 (96.4, 199.0)	27 (84)	118.9 (78.4, 173.1)	17 (53)	74.9 (43.6, 119.9)	5 (16)	22.0 (7.2, 51.4)	0 (0)	0 (0, 16.3)
2009	18	68.2 (40.5, 107.9)	16 (89)	60.7 (34.7, 98.5)	12 (67)	45.5 (23.5, 79.5)	4 (22)	15.2 (4.1, 38.8)	0 (0)	0 (0, 14.0)
2010	29	95.5 (63.9, 137.1)	29 (100)	95.5 (63.9, 137.1)	20 (69)	65.8 (40.2, 101.7)	11 (38)	36.2 (18.1, 64.8)	0 (0)	0 (0, 12.1)
2011	40	116.7 (83.3, 158.9)	36 (90)	105.0 (73.5, 145.4)	18 (45)	52.5 (31.1, 83.0)	14 (35)	40.8 (22.3, 68.5)	2 (5)	5.8 (0.7, 21.1)
2012	44	115.7 (84.1, 155.3)	39 (89)	102.6 (72.9, 140.2)	23 (52)	60.5 (38.3, 90.8)	9 (20)	23.7 (10.8, 44.9)	2 (5)	5.3 (0.6, 19.0)
2013	61	146.6 (112.1, 188.3)	59 (97)	141.8 (107.9, 182.9)	27 (44)	64.9 (42.8, 94.4)	21 (34)	50.5 (31.2, 77.1)	0 (0)	0 (0, 8.9)
2014	89	198.7 (159.5, 244.5)	88 (99)	196.4 (157.5, 242.0)	45 (51)	100.4 (73.3, 134.4)	31 (35)	69.2 (47.0, 98.2)	6 (7)	13.4 (4.9, 29.2)
2015	148	312.7 (264.4, 367.4)	140 (95)	295.8 (248.9, 349.1)	86 (58)	181.7 (145.4, 224.4)	26 (18)	54.9 (35.9, 80.5)	5 (3)	10.6 (3.4, 24.7)
2016	141	285.1 (240.0, 336.3)	132 (94)	266.9 (223.3, 316.5)	81 (57)	163.8 (130.1, 203.6)	25 (18)	50.6 (32.7, 74.6)	3 (2)	6.1 (1.3, 17.7)
2017	169	327.9 (280.4, 381.3)	157 (93)	304.7 (258.9, 356.2)	111 (67)	215.4 (177.2, 259.4)	47 (28)	91.2 (67.0, 121.3)	13 (8)	25.2 (13.4, 43.1)
2018	149	246.1 (208.1, 288.9)	144 (97)	237.8 (200.6, 280.0)	74 (50)	122.2 (96.0, 153.4)	67 (45)	110.6 (85.8, 140.5)	12 (8)	19.8 (10.2, 34.6)
Total	986	190.3 (178.6, 202.5)	923 (94)	178.1 (166.8, 190.0)	553 (56)	106.8 (98.1, 116.1)	272 (28)	52.6 (46.6, 59.2)	43 (4)	8.3 (6.0, 11.2)

[a]Denominator for drug-specific percentages = number of polysubstance-involved deaths for each corresponding year
[b]Per 100,000 person-years
Drug categories are not mutually exclusive

© 2022 Fine DR et al. *JAMA Network Open.*

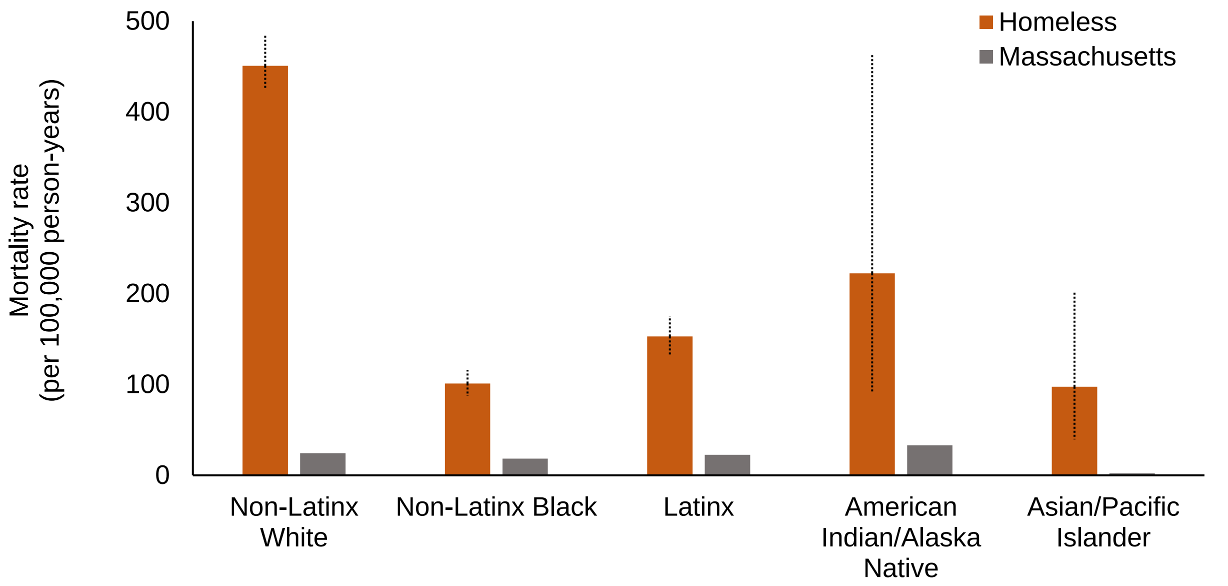

SRR[a] (95% CI)	18.6 (17.5, 19.9)	5.5 (4.8, 6.3)	6.7 (5.9, 7.7)	6.7 (2.7, 14.0)	44.3 (18.0, 92.2)

eFigure 1. Drug Overdose Mortality in the Boston Health Care for the Homeless Cohort, Stratified by Race and Ethnicity

White, Black, and Latinx mortality rates are age- and sex-standardized; American Indian/Alaska Native and Asian/Pacific Islander are only age-standardized due to cell suppression in CDC Wonder data.
[a]SRR=standardized rate ratio (calculated as standardized mortality rate in the BHCHP cohort divided by the standardized mortality rate in the general Massachusetts adult population).

© 2022 Fine DR et al. *JAMA Network Open.*

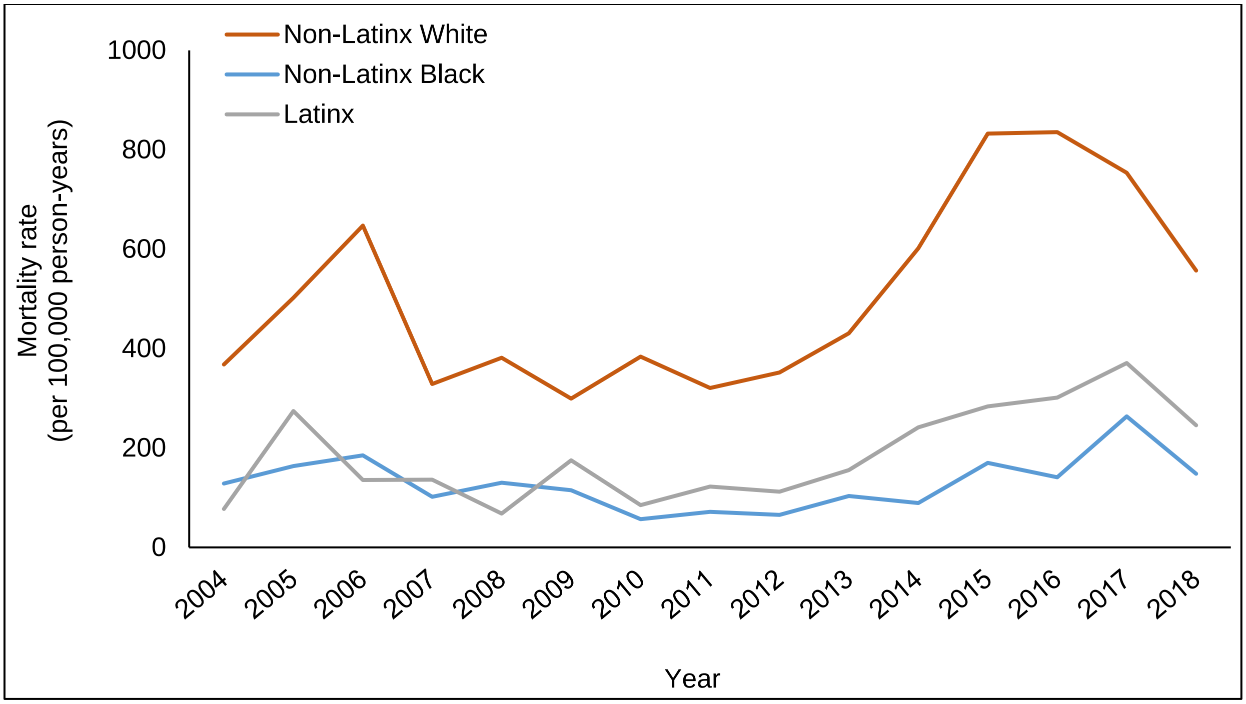

eFigure 2. Crude Drug Overdose Mortality in the Boston Health Care for the Homeless Cohort From 2004 to 2018, Stratified by Race and Ethnicity

© 2022 Fine DR et al. *JAMA Network Open.*

eTable 3. Opioid-Involved Overdose Mortality by Race and Ethnicity in the Boston Health Care for the Homeless Cohort

	Non-Latinx White		Non-Latinx Black		Latinx		
**Drug(s) implicated**	**Crude Rate[a] (95% CI)**	**Proportion**	**Crude Rate[a] (95% CI)**	**Proportion**	**Crude Rate[a] (95% CI)**	**Proportion**	**P value[b]**
All opioid	507.3 (477.8, 538.2)	--	101.8 (86.1, 119.4)	--	190.1 (163.8, 219.4)	--	--
Opioid alone	216.6 (197.4, 237.1)	0.43	38.0 (28.7, 49.3)	0.37	71.5 (55.5, 89.9)	0.37	0.23
Opioid + ≥ 1 other substance	290.7 (268.5, 314.3)	0.57	63.8 (51.5, 78.0)	0.63	118.9 (98.4, 142.5)	0.63	--
Opioid + cocaine	154.4 (138.3, 171.8)	0.53	46.1 (35.8, 58.5)	0.72	74.2 (58.2, 93.3)	0.62	<0.001
Opioid + alcohol	94.9 (82.4, 108.8)	0.33	27.8 (20.0, 37.7)	0.44	48.8 (36.0, 64.7)	0.41	0.009
Opioid + benzodiazepine	92.2 (78.8, 105.8)	0.32	4.7 (1.9, 9.8)	0.07	23.6 (14.8, 35.1)	0.20	<0.001
Opioid + psychostimulant	14.7 (10.0, 20.8)	0.05	1.4 (0.2, 4.9)	0.02	2.0 (0.2, 7.3)	0.02	0.27

[a]Per 100,000 person-years
[b]P values are based on Fisher exact tests assessing the differences in proportions

© 2022 Fine DR et al. *JAMA Network Open.*

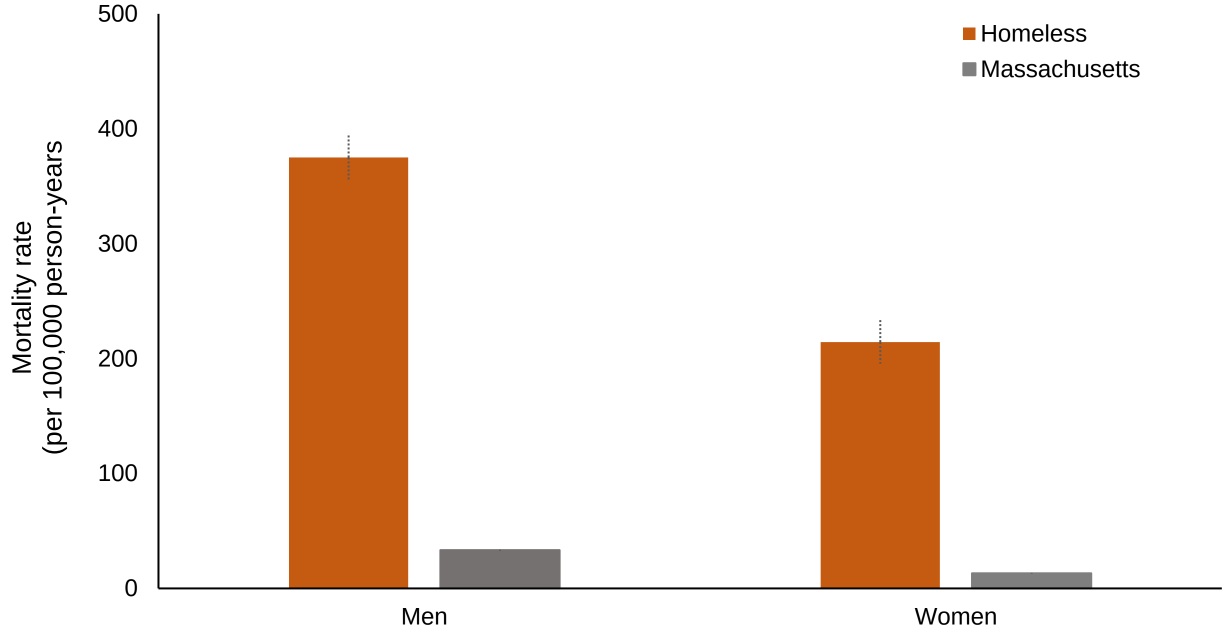

**SRR[a] (95% CI)**	11.3 (10.7, 11.9)	16.2 (14.8, 17.8)

eFigure 3. Drug Overdose Mortality in a Cohort of Homeless-Experienced Adults, Stratified by Sex

Mortality rates are age-standardized. [a]SRR=standardized rate ratio (calculated as standardized mortality rate in the BHCHP cohort divided by the standardized mortality rate in the general Massachusetts adult population).

© 2022 Fine DR et al. *JAMA Network Open.*

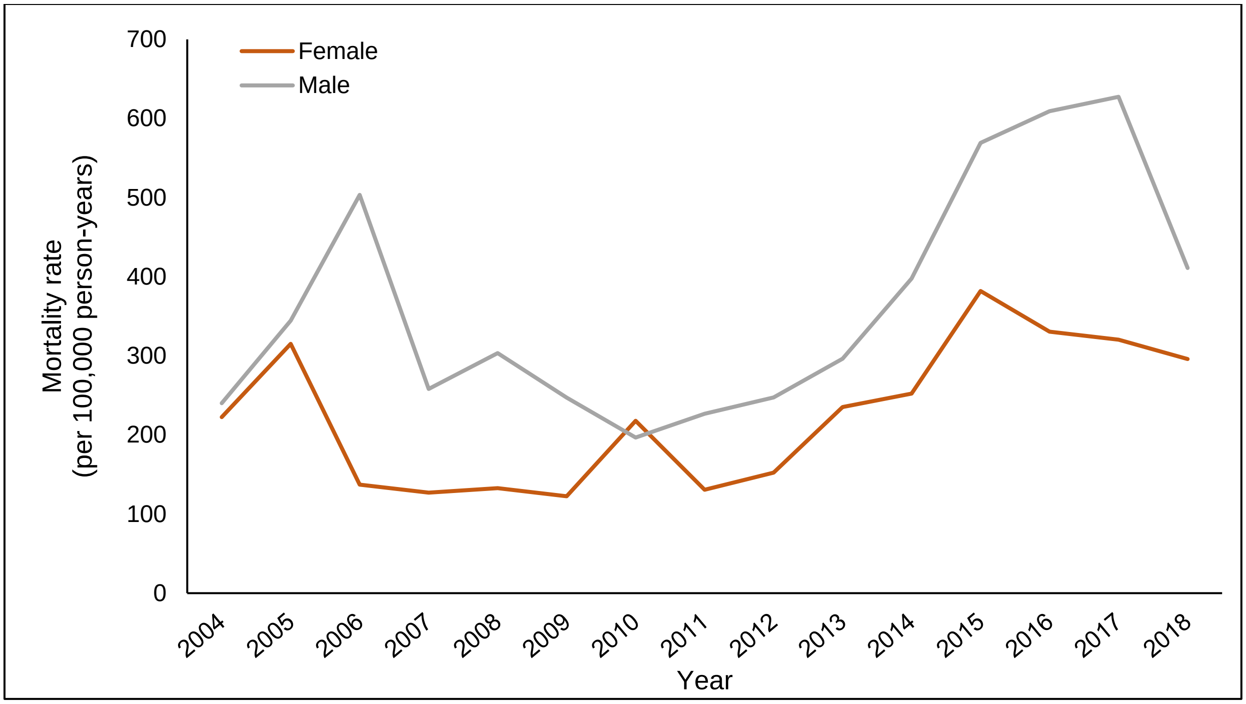

eFigure 4. Crude Drug Overdose Mortality in the Boston Health Care for the Homeless Cohort From 2004 to 2018, Stratified by Sex

© 2022 Fine DR et al. *JAMA Network Open.*

eTable 4. Opioid-Involved Overdose Mortality by Sex in the Boston Health Care for the Homeless Cohort

	Male		Female		
**Drug(s) implicated**	**Crude Rate[a] (95% CI)**	**Proportion**	**Crude Rate[a] (95% CI)**	**Proportion**	**P value[b]**
All opioid	352.7 (332.7, 373.5)	--	213.4 (193.1, 235.2)	--	--
Opioid alone	146.1 (133.3, 159.7)	0.41	84.8 (72.2, 99.0)	0.40	0.60
Opioid + ≥ 1 other substance	206.6 (191.4, 222.7)	0.59	128.9 (112.9, 145.7)	0.60	--
Opioid + cocaine	114.6 (103.4, 126.8)	0.55	77.5 (65.4, 91.0)	0.60	0.18
Opioid + alcohol	77.1 (67.9, 87.2)	0.37	31.6 (24.1, 40.7)	0.25	0.002
Opioid + benzodiazepine	51.4 (44.0, 59.8)	0.25	43.2 (34.4, 53.6)	0.34	0.009
Opioid + psychostimulant	9.1 (6.1, 13.0)	0.04	4.7 (2.2, 9.0)	0.04	0.85

[a]Per 100,000 person-years
[b]P values are based on Fisher exact tests assessing the differences in proportions

© 2022 Fine DR et al. *JAMA Network Open.*
